# No association of *TNFRSF1B *variants with type 2 diabetes in Indians of Indo-European origin

**DOI:** 10.1186/1471-2350-12-110

**Published:** 2011-08-17

**Authors:** Rubina Tabassum, Anubha Mahajan, Ganesh Chauhan, Om Prakash Dwivedi, Himanshu Dubey, Vasudha Sharma, Bratashree Kundu, Saurabh Ghosh, Nikhil Tandon, Dwaipayan Bharadwaj

**Affiliations:** 1 Genomics and Molecular Medicine Unit, CSIR-Institute of Genomics and Integrative Biology, Delhi- 110 007, India; 2 Human Genetics Unit, Indian Statistical Institute, Kolkata- 700 108, India; 3 Department of Endocrinology, All India Institute of Medical Sciences, New Delhi-110 029, India

## Abstract

**Background:**

There has been no systematic evaluation of the association between genetic variants of type 2 receptor for TNFα (TNFR2) and type 2 diabetes, despite strong biological evidence for the role of this receptor in the pathogenesis of this complex disorder. In view of this, we performed a comprehensive association analysis of *TNFRSF1B *variants with type 2 diabetes in 4,200 Indo-European subjects from North India.

**Methods:**

The initial phase evaluated association of seven SNPs viz. rs652625, rs496888, rs6697733, rs945439, rs235249, rs17883432 and rs17884213 with type 2 diabetes in 2,115 participants (1,073 type 2 diabetes patients and 1,042 control subjects). Further, we conducted replication analysis of three associated SNPs in 2,085 subjects (1,047 type 2 diabetes patients and 1,038 control subjects).

**Results:**

We observed nominal association of rs945439, rs235249 and rs17884213 with type 2 diabetes (*P *< 0.05) in the initial phase. Haplotype CC of rs945439 and rs235249 conferred increased susceptibility for type 2 diabetes [OR = 1.19 (95%CI 1.03-1.37), *P *= 0.019/*P*_perm _= 0.076] whereas, TG haplotype of rs235249 and rs17884213 provided protection against type 2 diabetes [OR = 0.83 (95%CI 0.72-0.95, *P *= 7.2 × 10^-3^/*P*_perm _= 0.019]. We also observed suggestive association of rs496888 with plasma hsCRP levels [*P *= 0.042]. However, the association of rs945439, rs235249 and rs17884213 with type 2 diabetes was not replicated in the second study population. Meta-analysis of the two studies also failed to detect any association with type 2 diabetes.

**Conclusions:**

Our two-stage association analysis suggests that *TNFRSF1B *variants are not the determinants of genetic risk of type 2 diabetes in North Indians.

## Background

Low-grade systemic inflammation plays a crucial role in the manifestation of type 2 diabetes [1]. Activation of TNF-TNFR axis in response to elevated Tumor Necrosis Factor α (TNFα) levels, a pro-inflammatory cytokine, can be speculated to contribute to the causation of type 2 diabetes. TNFα which is involved in varied processes such as inflammatory, immune and metabolic events, is over-expressed in adipose and muscle tissues of obese and type 2 diabetic subjects [2-4]. The metabolic activities of TNFα are mainly mediated through TNFR2 (type 2 receptor for TNFα), providing the inflammation-metabolic interface in type 2 diabetes.

Enhanced TNFR2 expression and soluble TNFR2 (sTNFR2) levels have been observed in obesity, insulin resistance and cardiovascular diseases [5-7]. While genetic variants of *TNFRSF1B *(TNFR2 gene) have been shown to be associated with various metabolic and inflammatory disorders [8-11], the investigations were limited to only two polymorphisms-M196R (rs1061622) and (CA)_n _repeat polymorphism. These two polymorphisms have not been analyzed extensively for their influence on the risk of type 2 diabetes. Only a solitary report suggested association of (CA)_n _repeat polymorphism of *TNFRSF1B *with diabetic neuropathy [9], while another study refuted its role in type 2 diabetes [12]. Moreover, the only study that investigated M196R failed to provide evidence for its association with type 2 diabetes [12]. Recently, we also investigated the association of these well-studied common polymorphisms along with 3'UTR rs3397 of *TNFRSF1B *with type 2 diabetes in North Indian population but did not find any significant association [13].

The inconsistent reports suggest that the role of *TNFRSF1B *variants in the pathogenesis of type 2 diabetes is not clear yet, necessitating comprehensive evaluation of *TNFRSF1B *variants. In view of this, we performed association analysis of seven SNPs of *TNFRSF1B *with type 2 diabetes in Indo-Europeans, followed by a replication analysis of three SNPs in an independent study population of Indo-European subjects from North India.

## Methods

### Subjects

A total of 4,200 unrelated Indo-European subjects including 2,120 patients with type 2 diabetes and 2,080 non-diabetic controls from two independent study populations from North India participated in the study. For the initial association analysis, we recruited 2,115 subjects comprising 1,073 patients with type 2 diabetes and 1,042 non-diabetic controls. Replication was assessed in an independent study population of 2,085 individuals including 1,047 type 2 diabetes patients and 1,038 control subjects. The subjects in both the study populations were recruited according to the inclusion and exclusion criteria described previously [14]. Briefly, patients with type 2 diabetes diagnosed according to World Health Organization criteria [15]. The non-diabetic control samples were collected by organizing 'Diabetes Awareness Camps' in the urban regions in and around Delhi from North India. Subjects of ≥40 years of age with no family history of diabetes in first and/or second degree relatives who had glycated haemoglobin (HbA1c) level ≤6.0% and fasting glucose level <110 mg/dL were recruited as controls. All the participants provided written informed consent. The study was approved by the Ethics Committees of the participating institutions and was in accordance with the principles of the Helsinki Declaration.

### Anthropometric and biochemical measurements

Anthropometric and biochemical parameters related to type 2 diabetes were measured in type 2 diabetic patients and control subjects. Height, weight, waist circumference and hip circumference were measured following standard guidelines. Body mass index (BMI) and waist-to-hip ratio (WHR) were calculated from these measurements. Levels of glucose, HbA1c, insulin, C-peptide, total cholesterol, triglycerides (TG), high-density lipoprotein cholesterol (HDL-C), low-density lipoprotein cholesterol (LDL-C), urea, uric acid and creatinine were measured as described earlier [14]. Levels of hsCRP were estimated using either ELISA (Biocheck Inc., CA, USA) or Cobas Integra 400 Plus (Roche Diagnostic, Mannheim, Germany). The homeostasis model assessment of insulin resistance (HOMA-IR) index was calculated as described earlier [16].

### SNP selection and Genotyping

We first determined linkage disequilibrium (LD) pattern and tag SNPs for *TNFRSF1B *using European population data (HapMap 2). Four tag SNPs or their proxy SNPs (prioritized based on their locations in functionally significant region) that captured most of the SNPs in the gene (rs496888, rs6697733, rs945439 and rs235249) were selected. One tag SNP (rs3397) had already been genotyped in same sample set in our previous study [13]. Tag SNPs not capturing other SNPs were avoided; instead SNPs in functionally significant regions (promoter and coding regions) considering spacing between the SNPs to cover the entire gene were preferred (rs652625, rs17883432 and rs17884213). With the present seven SNPs and two SNPs (rs1061622 and rs3397) from our previous study, we captured 63% of all the variants reported in HapMap for Caucasian population. A pictorial representation of the selected SNPs along with gene structure and LD pattern around *TNFRSF1B *in GIH (Gujarati Indians in Houston, Texas) population from HapMap data is provided in Additional file 1.

We genotyped seven SNPs-rs652625 (5' flank), rs496888 (intron 1), rs6697733 (intron 1), rs945439 (exon 2), rs235249 (intron 8), rs17883432 (exon 9) and rs17884213 (intron 9) for the initial association analysis. Genotyping was performed using GoldenGate assay on Illumina platform (Illumina Inc., San Diego, CA, USA). The genotyping data obtained was subjected to extensive quality control as provided in detail earlier [17]. Two SNPs (rs235249 and rs17883432) failed in Illumina assay and were genotyped using iPLEX on a MassARRAY System (Sequenom, San Deigo, CA, USA). The SNPs that passed Illumina quality control had an average call rate of 99% with concordance of >99.9% in genotype calls in 7% duplicates. The average genotyping success rate for SNPs genotyped on Sequenom was >95% with 100% concordance rate in 4% duplicates. In the replication phase, genotyping of rs945439, rs235249 and rs17884213 was performed using iPLEX on a MassARRAY System (Sequenom). These SNPs had average call rate of 96% with 100% consistency of genotype calls in 5% duplicates.

### Statistical Analysis

The statistical analyses were mainly performed using PLINK v. 1.07 [http://pngu.mgh.harvard.edu/~purcell/plink; [18]] or otherwise specified. Deviation from Hardy Weinberg Equilibrium (HWE) was tested using χ^2 ^analysis. Association of SNPs with type 2 diabetes was assessed using logistic regression analysis adjusted for age, sex and BMI. Meta-analysis was performed by combining the summary estimates (OR and 95%CI) both under random and fixed effect models. Association between genotypes and quantitative traits were determined only in control subjects using Kruskal-Wallis test using SPSS version 17.0. Pairwise LD between the SNPs was determined using Haploview 4.0 and the haplotype blocks were defined using the method of Gabriel et al., 2002 as implemented in Haploview V4.0 [19]. Haplotype association analysis adjusted for age, sex and BMI were carried out at 10,000 permutations. A *P *value < 0.017 was considered significant after Bonferroni correction (*P *= 0.05/3) in the replication analysis. Statistical power of the study was determined using Quanto software (http://hydra.usc.edu/gxe/) assuming log additive model of inheritance and 10% prevalence of type 2 diabetes at α = 0.05. The initial and combined phase of the study had 82% and 98% power respectively to detect the association of a SNP with OR of 1.25 and allele frequency of 0.19 (the least allele frequency for the present study).

## Results

The anthropometric and biochemical characteristics of patients with type 2 diabetes and control subjects in two study populations are provided in Table 1. Of the seven SNPs, rs652625 and rs17883432 had minor allele frequency (MAF) <5.0% and were excluded from subsequent association analysis. The genotypic distributions of the polymorphisms were in accordance with HWE both among type 2 diabetes patients and controls (all *P *> 0.047) (see Additional file 2).

**Table 1:** Anthropometric and clinical characteristics of the study populations

	Initial phase		Replication phase	
	
Characteristics	Type 2 diabetes patients	Control Subjects	Type 2 diabetes patients	Control subjects
N (Men/Women)	1019 (592/427)	1006 (606/400)	1047 (619/428)	1038 (516/522)
Age (years)	53 (45-62)	50 (44-60)	55 (49-62)	54 (45-64)
BMI (Kg/m^2^)				
Men	23.8 (22.0-26.0)	23.1 (20.1-25.7)	24.8 (22.7-27.8)	24.7 (22.3-27.4)
Women	26.7 (24.2-29.2)	25.0 (21.1-28.5)	27.4 (24.6-30.6)	26.4 (23.1-29.3)
WHR				
Men	1.00 (0.97-1.03)	0.97 (0.92-1.0)	0.98 (0.95-1.03)	0.97 (0.93-1.01)
Women	1.00 (0.97-1.03)	0.86 (0.82-0.92)	0.93 (0.87-0.97)	0.86 (0.82-0.90)
Systolic BP (mmHg)	130 (130-140)	120 (112-132)	130 (122-140)	130 (120-140)
Diastolic BP (mmHg)	80 (78-90)	80 (70-88)	82 (80-90)	80 (78-90)
HbA1c (%)	7.8 (6.5-9.4)	5.2 (4.9-5.6)	8.2 (6.9-9.6)	5.6 (5.3-5.9)
FPG (mmol/L)	7.9 (6.4-10.3)	4.9 (4.5-5.3)	7.8 (6.2-10.3)	4.9 (4.4-5.3)
FPI (pmol/L)	82.8 (42.0-166.8)	32.4 (17.4-57.6)	74.4 (26.0-96.0)	43.8 (28.2-63.6)
HOMA-IR	5.20 (2.30-9.60)	1.20 (0.60-2.00)	4.30 (2.30-9.30)	1.60 (1.00-2.40)
C-peptide (nmol/L)	0.89 (0.56-1.36)	0.53 (0.36-0.73)	1.05 (0.69-1.60)	0.66 (0.50-0.86)
hsCRP (mg/L)	2.20 (0.90-4.70)	1.30 (0.60-3.00)	1.86 (0.90-3.44)	1.61 (0.90-3.04)
TC (mmol/L)	4.20 (3.50-5.00)	4.40 (3.7-5.10)	4.64 (3.86-5.42)	4.91 (4.22-5.52)
LDL-C (mmol/L)	2.57 (1.99-3.36)	2.79 (2.33-3.41)	2.73 (2.10-3.42)	3.01 (2.49-3.51)
HDL-C (mmol/L)	1.03 (0.88-1.22)	1.06 (0.88-1.28)	1.11 (0.94-1.34)	1.24 (1.06-1.46)
TG (mmol/L)	1.60 (1.10-2.20)	1.30 (1.00-1.80)	1.43 (0.98-2.13)	1.22 (0.86-1.64)

Data are median values with interquartile ranges in parentheses. WHR: waist-hip ratio; FPG: fasting plasma glucose; FPI: fasting plasma insulin; HOMA-IR: homeostasis model assessment of insulin resistance; TC: total cholesterol; TG: triglyceride

In the initial phase, we observed nominal association of three SNPs- rs945439, rs235249 and rs17884213 with type 2 diabetes at *P *< 0.05 (Table 2). Stronger association was observed under recessive model for rs17884213 and rs235249. Individuals who were homozygous for minor allele of rs17884213 and rs235249 had OR of 1.80 (95%CI 1.25-2.60, *P *= 1.7 × 10^-3^) and 1.69 (95%CI 1.17-2.46, *P *= 5.7 × 10^-3^) respectively, under recessive model. Other genetic variants studied here-rs496888 and rs6697733 both in intron 1, did not show association with type 2 diabetes in the initial study population.

**Table 2:** Association analysis of *TNFRSF1B *SNPs with type 2 diabetes in Indo-European population from North India

	Initial phase				Replication phase				Meta-analysis	
	
	Genotype distribution				Genotype distribution					
	
SNP	Type 2 diabetes patients	Control Subjects	OR (95%CI)	*P*	Type 2 diabetes patients	Control Subjects	OR (95%CI)	*P*	OR^r^/ OR^f^	*P *^r^/ P^f^
rs496888 (A/G)* (Intron 1)	669 (66.0) 299 (29.6) 45 (4.4)	642 (64.5) 314 (31.5) 40 (4.0)	0.95 (0.81-1.11)	0.484						
rs6697733 (T/C)* (Intron 1)	536 (52.8) 404 (39.7) 76 (7.5)	544 (54.1) 400 (39.9) 61 (6.0)	1.07 (0.93-1.24)	0.346						
rs945439 (T/C) (Exon 2)	540 (53.3) 395 (38.8) 80 (7.9)	566 (56.3) 386 (38.5) 53 (5.3)	1.17 (1.01-1.36)	**0.036**	546 (54.0) 393 (38.9) 72 (7.1)	523 (52.2) 404 (40.3) 75 (7.5)	0.94 (0.81-1.08)	0.364	1.05/1.04	0.686/0.420
rs235249 (T/C) (Intron 8)	503 (51.6) 392 (40.2) 80 (8.2)	545 (55.6) 385 (39.2) 51 (5.2)	1.22 (1.05-1.41)	**0.010**	561 (54.6) 391 (38.1) 75 (7.3)	536 (53.1) 399 (39.5) 75 (7.4)	0.95 (0.82-1.09)	0.440	1.07/1.06	0.583/0.227
rs17884213 (G/A) (Intron 9)	550 (54.3) 376 (37.0) 88 (8.7)	564 (56.1) 390 (38.9) 50 (5.0)	1.17 (1.02-1.36)	**0.031**	569 (56.7) 373 (37.1) 62 (6.2)	525 (53.0) 407 (41.1) 58 (5.9)	0.92 (0.80-1.07)	0.289	1.04/1.04	0.733/0.432

Association was assessed assuming additive model adjusted for age, sex and BMI. The significant associations are highlighted in bold text. OR: odds ratio; CI: confidence interval; OR^r^: odds ratio for random effects meta-analysis; OR^f^: odds ratio for fixed effects meta-analysis. * SNPs not genotyped in the replication phase.

**Figure 1:**
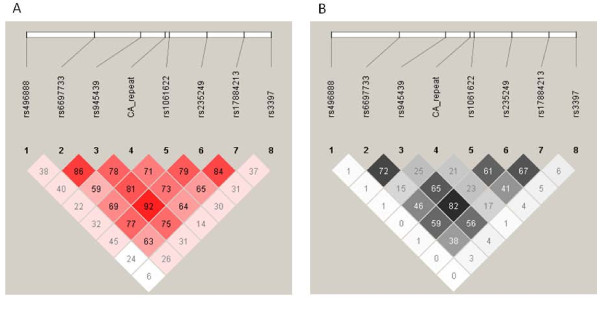
**Pairwise linkage disequilibrium between the selected SNPs in *TNFRSF1B***. (A) Plot with D' values; (B) Plot with r^2^values between the SNPs.

SNP rs945439 was found to be in LD with rs235249 (D' = 0.92 and r^2 ^= 0.83) (Figure 1). The haplotype CC containing minor alleles of these two SNPs was associated with increased susceptibility for type 2 diabetes with OR of 1.19 (95%CI 1.03-1.37, *P *= 0.019/*P*_perm _= 0.076) (Table 3). We also observed some extent of LD between rs235249 and rs17884213 (D' = 0.83, r^2 ^= 0.66). Haplotype TG encompassing major alleles of rs235249 and rs17884213 was associated with protection against type 2 diabetes with OR of 0.83 (95%CI 0.72-0.95, *P *= 7.2 × 10^-3^). The association remained significant after performing 10,000 permutations (*P*_perm _= 0.019).

**Table 3:** Association of haplotypes of *TNFRSF1B *with type 2 diabetes in North Indian population

		Initial phase				Replication phase			
		
SNPs	Haplotype	Type 2 diabetes patients	Control Subjects	OR (95%CI)	*P* *P*_perm_	Type 2 diabetes patients	Control Subjects	OR (95%CI)	*P* *P*_perm_
rs235249 rs17884213	TG	0.683	0.721	0.83 (0.72-0.95)	7.6 × 10^-3^ 0.021	0.701	0.688	1.05 (0.92-1.20)	0.354 0.743
rs945439 rs235249	CC	0.261	0.230	1.19 (1.03-1.37)	0.019 0.076	0.247	0.259	0.93 (0.81-1.07)	0.353 0.755

OR: odds ratio; CI: confidence interval; *P*_perm_: *P *values obtained after performing 10,000 permutations.

We then investigated the effect of *TNFRSF1B *polymorphisms on quantitative metabolic traits related to type 2 diabetes viz BMI, WHR, HbA1c, fasting glucose, insulin, C-peptide, total cholesterol, TG, HDL-C, LDL-C, urea, uric acid and creatinine (Table 4). For this, the clinical variables of only control subjects were compared across the genotypes of the SNPs. We observed suggestive association of rs496888 with plasma hsCRP levels (*P *= 0.042). No influence of other variants on any of the metabolic traits investigated was found.

**Table 4:** Association analysis of SNPs with quantitative traits in control subjects in the initial phase

	rs496888		rs6697733		rs945439		rs235249		rs17884213	
	
	Median (IQR)	*P*	Median (IQR)	*P*	Median (IQR)	*P*	Median (IQR)	*P*	Median (IQR)	*P*
	
Trait	AA/AG/GG		TT/TC/CC		TT/TC/CC		TT/TC/CC		GG/GA/AA	
BMI	23.7 (20.4-27.0) 23.5 (20.0-26.6) 24.6 (22.4-29.0)	0.132	23.9 (20.5-26.9) 23.6 (20.2-26.9) 24.1 (20.2-27.6)	0.593	23.7 (20.4-26.7) 23.6 (20.3-27.0) 25.0 (21.8-28.0)	0.209	23.7 (20.4-26.9) 23.5 (20.3-26.9) 25.0 (21.7-27.8)	0.268	23.9 (20.4-27.2) 23.4 (20.3-26.4) 25.5 (21.6-28.2)	0.063
WHR	0.93 (0.87-0.98) 0.92 (0.86-0.98) 0.93 (0.87-0.98)	0.913	0.93 (0.87-0.98) 0.93 (0.87-0.99) 0.92 (0.86-0.97)	0.549	0.93 (0.87-0.98) 0.93 (0.86-0.99) 0.91 (0.87-0.97)	0.446	0.93 (0.87-0.98) 0.93 (0.86-0.99) 0.91 (0.87-0.97)	0.582	0.93 (0.86-0.98) 0.93 (0.87-0.99) 0.93 (0.87-0.97)	0.568
HbA1c	5.2 (4.9-5.6) 5.2 (4.9-5.6) 5.2 (4.9-5.3)	0.358	5.3 (4.9-5.6) 5.2 (4.9-5.5) 5.2 (4.8-5.5)	0.174	5.3 (4.9-5.6) 5.2 (4.9-5.5) 5.2 (4.8-5.5)	0.247	5.3 (4.9-5.6) 5.2 (4.5-5.5) 5.3 (4.9-5.5)	0.472	5.3 (4.9-5.6) 5.2 (4.9-5.6) 5.1 (4.9-5.5)	0.194
FPG	4.9 (4.5-5.3) 4.9 (4.5-5.2) 5.0 (4.3-5.3)	0.425	5.0 (4.5-5.3) 4.9 (4.5-5.2) 4.9 (4.5-5.2)	0.348	4.9 (4.5-5.3) 4.9 (4.5-5.2) 4.9 (4.5-5.2)	0.315	4.9 (4.5-5.3) 4.9 (4.5-5.2) 5.0 (4.6-5.2)	0.275	4.9 (4.5-5.3) 4.9 (4.5-5.2) 5.0 (4.6-5.2)	0.236
FPI	32.4 (16.8-56.4) 31.8 (18.6-60.0) 36.0 (21.0-67.2)	0.543	33.6 (17.4-61.2) 31.8 (18.0-54.0) 26.4 (16.2-43.2)	0.279	33.6 (17.4-60.6) 31.8 (18.6-54.0) 24.6 (15.6-41.4)	0.190	32.4 (16.8-59.4) 31.2 (18.6-54.0) 30.6 (15.6-51.0)	0.890	34.2 (18.0-59.4) 30.6 (17.4-54.0) 30.6 (17.4-44.4)	0.536
HOMA-IR	1.2 (0.6-2.0) 1.1 (0.6-2.0) 1.1 (0.7-2.4)	0.717	1.2 (0.6-2.2) 1.2 (0.6-1.9) 1.0 (0.6-1.5)	0.227	1.2 (0.6-2.2) 1.2 (0.6-1.9) 0.9 (0.5-1.4)	0.200	1.1 (0.6-2.1) 1.2 (0.6-1.9) 1.1 (0.6-1.9)	0.896	1.2 (0.6-2.2) 1.1 (0.6-1.9) 1.1 (0.6-1.5)	0.553
C-peptide	0.53 (0.36-0.73) 0.53 (0.40-0.76) 0.60 (0.43-0.79)	0.445	0.53 (0.40-0.76) 0.53 (0.36-0.73) 0.50 (0.33-0.63)	0.278	0.53 (0.40-0.76) 0.53 (0.36-0.73) 0.50 (0.33-0.60)	0.158	0.53 (0.40-0.76) 0.53 (0.36-0.76) 0.50 (0.33-0.63)	0.596	0.53 (0.40-0.76) 0.53 (0.36-0.73) 0.50 (0.40-0.66)	0.716
CRP	1.25 (0.59-2.67) 1.11 (0.58-2.57) 0.77 (0.37-1.64)	**0.042**	1.20 (0.58-2.48) 1.14 (0.58-2.86) 1.13 (0.66-2.78)	0.860	1.21 (0.57-2.52) 1.13 (0.59-2.87) 1.13 (0.70-2.54)	0.855	1.19 (0.55-2.48) 1.16 (0.60-2.93) 1.14 (0.75-2.84)	0.894	1.28 (0.57-2.70) 1.07 (0.57-2.43) 1.21 (0.71-3.35)	0.212
TC	4.42 (3.80-5.17) 4.42 (3.78-5.09) 4.14 (3.70-4.86)	0.408	4.42 (3.83-5.15) 4.37 (3.75-5.12) 4.34 (3.72-5.04)	0.605	4.4 (3.80-5.17) 4.37 (3.72-5.09) 4.42 (3.93-5.20)	0.532	4.4 (3.78-5.15) 4.4 (3.75-5.12) 4.4 (3.90-5.09)	0.986	4.40 (3.78-5.15) 4.42 (3.80-5.12) 4.27 (3.85-4.84)	0.636
HDL-C	1.06 (0.88-1.29) 1.06 (0.91-1.29) 1.09 (0.93-1.19)	0.857	1.06 (0.91-1.29) 1.06 (0.88-1.29) 1.11 (0.85-1.34)	0.947	1.06 (0.91-1.29) 1.06 (0.88-1.29) 1.09 (0.88-1.27)	0.899	1.06 (0.88-1.29) 1.06 (0.88-1.29) 1.06 (0.88-1.27)	0.938	1.06 (0.91-1.29) 1.09 (0.88-1.29) 1.03 (0.85-1.22)	0.637
LDL-C	2.82 (2.35-3.44) 2.79 (2.35-3.36) 2.66 (2.17-3.41)	0.775	2.82 (2.38-3.47) 2.74 (2.25-3.39) 2.77 (2.43-3.44)	0.338	2.82 (2.35-3.47) 2.74 (2.25-3.39) 2.74 (2.51-3.44)	0.293	2.82 (2.35-3.47) 2.79 (2.30-3.41) 2.74 (2.48-3.31)	0.814	2.79 (2.35-3.41) 2.84 (2.33-3.41) 2.64 (2.28-3.26)	0.699
TG	1.32 (0.98-1.84) 1.34 (0.98-1.83) 1.33 (1.05-1.77)	0.951	1.33 (0.98-1.86) 1.31 (0.96-1.76) 1.38 (1.11-1.73)	0.562	1.32 (0.97-1.9) 1.31 (0.97-1.74) 1.36 (1.11-1.73)	0.578	1.32 (0.96-1.86) 1.37 (1.01-1.76) 1.36 (1.11-1.81)	0.839	1.32 (0.96-1.86) 1.31 (0.99-1.77) 1.43 (1.06-1.75)	0.940

IQR: interquartile range. The *P *values were obtained by performing Kruskal-Wallis test.

Further, we attempted to replicate the associations of rs945439, rs235249 and rs17884213 in an independent study population of 2,085 Indo-European subjects. We did not find significant association of any of these SNPs with type 2 diabetes in the replication study population (*P *= 0.364, 0.440 and 0.289 respectively). We also performed meta-analysis of two studies that failed to identify any association with type 2 diabetes (Table 2).

## Discussion

India has the largest number of individuals suffering from type 2 diabetes, with the prevalence likely to increase to approximately 80 million over the next two decades [20]. The factors contributing to such a high risk of type 2 diabetes in this part of world are not clearly understood yet. Previously, we demonstrated role of inflammation in the development of type 2 diabetes in Indo-European population [21]. Our group also showed association of *TNF-LTA *locus variants with type 2 diabetes in this population [22]. This suggests *TNFRSF1B *as an important biological candidate gene for type 2 diabetes in Indo-Europeans.

Previously, we investigated association of three *TNFRSF1B *polymorphisms- rs1061622 (M196R; exon6), rs3397 (3'UTR) and (CA)_n _and demonstrated that these polymorphisms are not associated with type 2 diabetes or its complications in Indo-Europeans [13]. Here, we carried out a comprehensive association analysis of *TNFRSF1B *variants with type 2 diabetes in Indo-Europeans.

In the initial phase, we observed nominal association of rs235249 in intron 8 and rs17884213 in intron 9 with type 2 diabetes. Also, haplotype TG, harboring major alleles of these two SNPs was associated with protection against type 2 diabetes. SNP rs945439, a synonymous variation in exon 2 (K56K), also showed nominal association with type 2 diabetes in the initial phase. In spite of being a coding variation, this SNP had never been evaluated for its role in the manifestation of any metabolic disorder. SNPs rs496888 and rs6697733 both in intron 1, did not influence the susceptibility to type 2 diabetes in the study population.

The investigation of the association of genetic variants of *TNFRSF1B *with quantitative traits revealed only the nominal association of rs496888 with hsCRP levels. Hence, being a pro-inflammatory gene, we did observe a nominal association with inflammation. No association of other variants with any other metabolic traits suggests that either these associated SNPs have very small effect on the quantitative traits analyzed here or they might modulate the risk of type 2 diabetes by influencing some other biochemical traits not investigated here.

The observed differences in the results of initial and replication phases might reflect the heterogeneity in the population. However, subjects in both the study populations have been collected with great caution regarding ethnicity and geographical regions and form a homogenous cluster as reported by the Indian Genome Variation Consortium [23]. This strategy though cannot completely rule out but decreases the effects of population stratification. Importantly, a study by Rosenberg *et al *[24] has stated that false positives arising due to genetic heterogeneity in the diverse Indian population could be smaller than expected. It is pertinent to mention here that in our recent study we successfully replicated the association of top eight GWAS confirmed loci with type 2 diabetes in the same population [17].

Further, to determine the structure of our population, we performed a multidimensional scaling based on 608 unlinked markers genotyped in our initial study population which clearly shows that the study population belongs to one cluster (see Additional file 3). We have also adjusted the associations for principal components obtained from an ongoing genome wide association study on the same study population and found that the associations are not significant in either of the study population, merging the two study populations or after meta-analysis (data not presented). To take care of the difference in allele frequency of rs945439, we have meta-analyzed the data of the two study populations both under random and fixed effect models, instead of combining the two data sets.

## Conclusion

The present two-stage association analysis did not detect any association between type 2 diabetes and *TNFRSF1B *genetic variants in Indo-Europeans from North India. The results are consistent with our earlier observation of no association of well-studied polymorphisms with type 2 diabetes in Indo-European population. Thus, we conclude, *TNFESF1B *is not a major genetic risk factor for type 2 diabetes in Indo-Europeans from North India.

## Competing interests

The authors declare that they have no competing interests.

## Authors' contributions

RT designed and processed the study, interpreted the data and wrote the first draft of the manuscript. AM contributed in the data interpretation and manuscript writing. OPD and GC contributed in genotyping and manuscript writing. VS and BK did sample management and DNA purification. SG helped in the statistical analysis of the data. DB and NT conceived and supervised the study and critically evaluated the study and manuscript. All the authors read and approved the final manuscript.

## Pre-publication history

The pre-publication history for this paper can be accessed here:

http://www.biomedcentral.com/1471-2350/12/110/prepub

## Supplementary Material

Additional file 1 **Schematic presentation of selection of SNPs for the study and linkage disequilibrium pattern around TNFRSF1B gene in GIH population**. A figure described the linkage disequllibrium between the SNPs, drawn using the GIH population data from HapMap 3. The arrow above the SNPs in LD plot shows the SNPs that have been genotyped. * These SNPs were genotyped in our previous study (Ref). Arrows with dotted pattern lines shows the SNPs not presented in LD plot as these were not available in HapMap 3. Click here for file

Additional file 2 **Hardy Weinberg Equilibrium in cases and control subjects in initial phase and replication phase for all the SNPs**. A table summarized the minor allele frequencies and Hardy Weinberg Equilibrium of the SNPs in cases and control subjects. MAF: Minor allele frequency; P_HWE: P value for Hardy Weinberg Equilibrium; NA: Not genotyped in replication phase. Click here for file

Additional file 3 **³Multidimensional scaling based on markers genotyped in our initial study²**. A figure presented the multidimensional scaling based on 608 unlinked markers (r2 < 0.20) that were used to obtain the positions on the first and second dimensions using PLINK for the samples of the initial phase. Click here for file
