# Supplementary figures and images for: No association of TNFRSF1B variants with type 2 diabetes in Indians of Indo-European origin
Source: BMC Med Genet. 2011 Aug 17;12:110. doi: 10.1186/1471-2350-12-110 (PMC3179441; doi:10.1186/1471-2350-12-110)

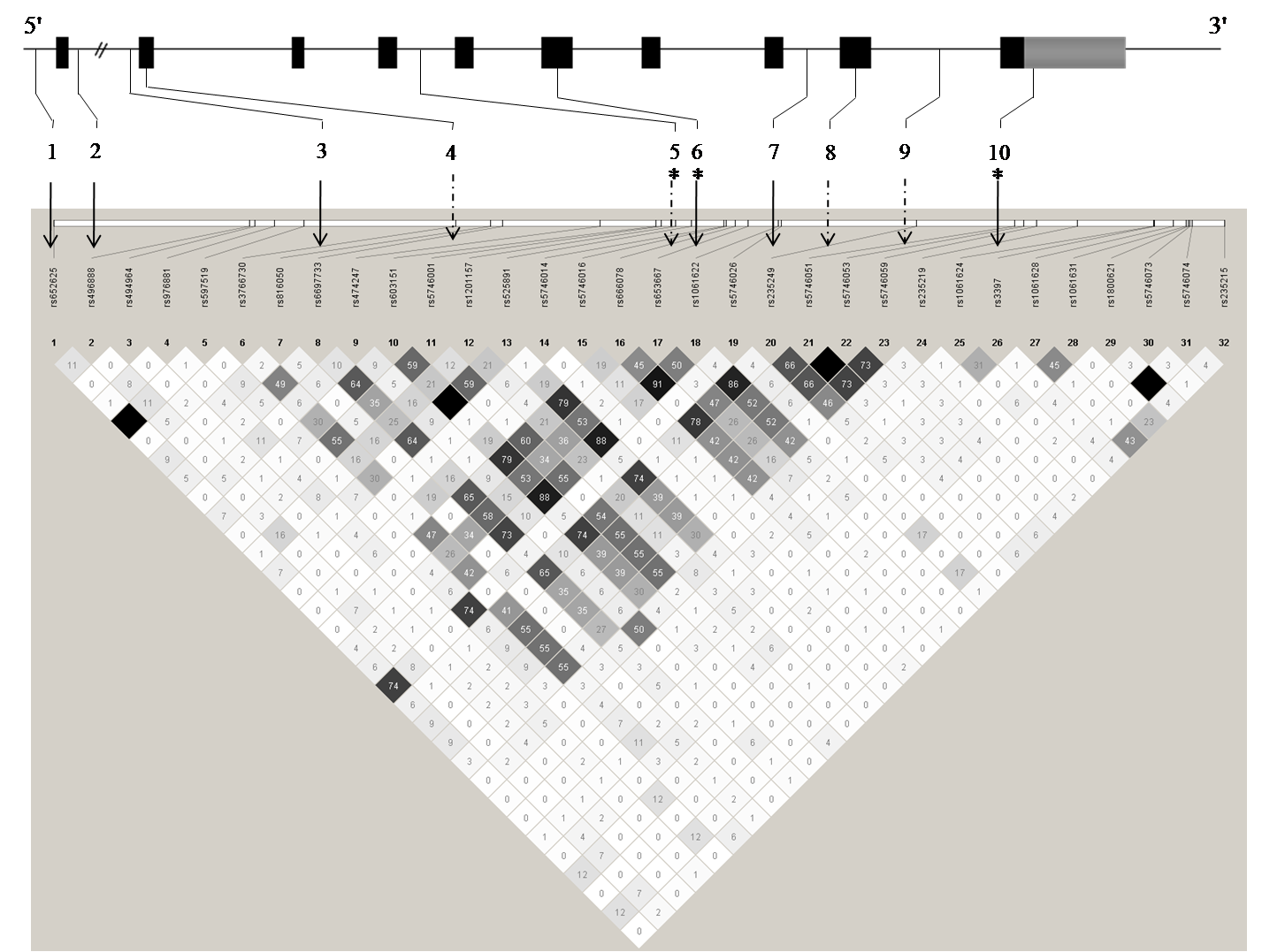

Supplement: Additional file 1 — Schematic presentation of selection of SNPs for the study and linkage disequilibrium pattern around TNFRSF1B gene in GIH population. A figure described the linkage disequllibrium between the SNPs, drawn using the GIH population data from HapMap 3. The arrow above the SNPs in LD plot shows the SNPs that have been genotyped. * These SNPs were genotyped in our previous study (Ref). Arrows with dotted pattern lines shows the SNPs not presented in LD plot as these were not available in HapMap 3. [file 1471-2350-12-110-S1.TIFF]

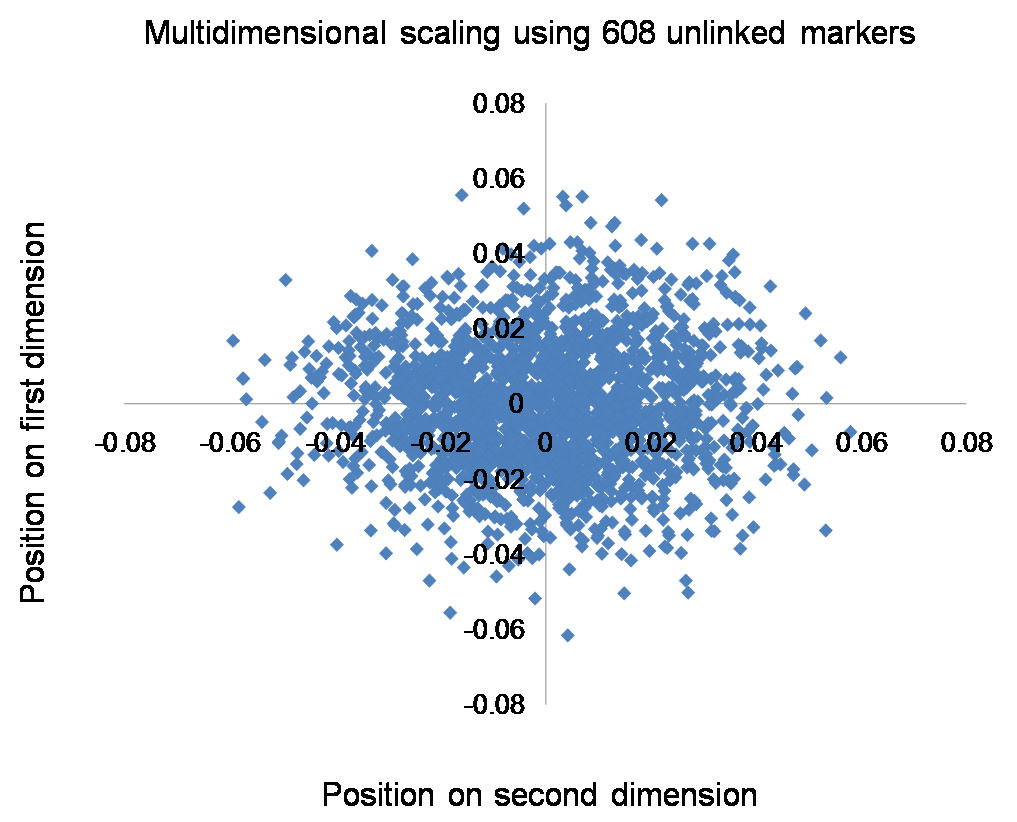

Supplement: Additional file 3 — ³Multidimensional scaling based on markers genotyped in our initial study². A figure presented the multidimensional scaling based on 608 unlinked markers (r2 < 0.20) that were used to obtain the positions on the first and second dimensions using PLINK for the samples of the initial phase. [file 1471-2350-12-110-S3.TIFF]
