# Supplementary material for: No association of TNFRSF1B variants with type 2 diabetes in Indians of Indo-European origin
Source: BMC Med Genet. 2011 Aug 17;12:110. doi: 10.1186/1471-2350-12-110 (PMC3179441; doi:10.1186/1471-2350-12-110)
Supplement: Additional file 2 — Hardy Weinberg Equilibrium in cases and control subjects in initial phase and replication phase for all the SNPs. A table summarized the minor allele frequencies and Hardy Weinberg Equilibrium of the SNPs in cases and control subjects. MAF: Minor allele frequency; P_HWE: P value for Hardy Weinberg Equilibrium; NA: Not genotyped in replication phase. [file 1471-2350-12-110-S2.DOC]

**Additional file 2:** Hardy Weinberg Equilibrium in cases and control subjects in initial phase and replication phase for all the SNPs

| **SNP** | **Initial phase** | | | | **Replication phase** | | | |
| --- | --- | --- | --- | --- | --- | --- | --- | --- |
|  | **Cases** | | **Controls** | | **Cases** | | **Controls** | |
|  | **MAF** | **P_HWE** | **MAF** | **P_HWE** | **MAF** | **P_HWE** | **MAF** | **P_HWE** |
| rs496888 | 0.192 | 0.128 | 0.198 | 0.842 | NA | NA | NA | NA |
| rs6697733 | 0.274 | 1.0 | 0.260 | 0.290 | NA | NA | NA | NA |
| rs945439 | 0.273 | 0.527 | 0.245 | 0.233 | 0.266 | 0.940 | 0.276 | 0.870 |
| rs235249 | 0.283 | 0.753 | 0.248 | 0.123 | 0.263 | 0.570 | 0.272 | 0.940 |
| rs17884213 | 0.272 | 0.047 | 0.244 | 0.104 | 0.247 | 0.932 | 0.264 | 0.086 |

MAF: Minor allele frequency; P_HWE: P value for Hardy Weinberg Equilibrium; NA: Not genotyped in replication phase
